# Magnetic Resonance Spectroscopy discriminates the response to microglial stimulation of wild type and Alzheimer’s disease models

**DOI:** 10.1038/srep19880

**Published:** 2016-01-27

**Authors:** Marie-Christine Pardon, Maria Yanez Lopez, Ding Yuchun, Małgorzata Marjańska, Malcolm Prior, Christopher Brignell, Samira Parhizkar, Alessandra Agostini, Li Bai, Dorothee P. Auer, Henryk M Faas

**Affiliations:** 1 Neuroscience, School of Life Sciences, University of Nottingham United Kingdom; 2 Sir Peter Mansfield Imaging Centre, School of Medicine, University of Nottingham United Kingdom; 3 School of Computer Sciences, University of Nottingham United Kingdom; 4 Center for Magnetic Resonance Research and Department of Radiology, University of Minnesota, Minneapolis, Minnesota, USA; 5 Medical Imaging Unit, School of Medicine, University of Nottingham United Kingdom; 6 School of Mathematics, University of Nottingham, United Kingdom

## Abstract

Microglia activation has emerged as a potential key factor in the pathogenesis of Alzheimer’s disease. Metabolite levels assessed by magnetic resonance spectroscopy (MRS) are used as markers of neuroinflammation in neurodegenerative diseases, but how they relate to microglial activation in health and chronic disease is incompletely understood. Using MRS, we monitored the brain metabolic response to lipopolysaccharides (LPS)-induced microglia activation *in vivo* in a transgenic mouse model of Alzheimer’s disease (APP/PS1) and healthy controls (wild-type (WT) littermates) over 4 hours. We assessed reactive gliosis by immunohistochemistry and correlated metabolic and histological measures. In WT mice, LPS induced a microglial phenotype consistent with activation, associated with a sustained increase in macromolecule and lipid levels (ML9). This effect was not seen in APP/PS1 mice, where LPS did not lead to a microglial response measured by histology, but induced a late increase in the putative inflammation marker myoinositol (mI) and metabolic changes in total creatine and taurine previously reported to be associated with amyloid load. We argue that ML9 and mI distinguish the response of WT and APP/PS1 mice to immune mediators. Lipid and macromolecule levels may represent a biomarker of activation of healthy microglia, while mI may not be a glial marker.

Microglial cells represent the endogenous defence and immune system in the brain, responsible for protecting the central nervous system against various types of pathogenic factors. While generally considered to be protective, they can adopt, when stimulated, diverse phenotypes from pro-inflammatory M1 to immunosuppressive M2 phenotypes, producing either cytotoxic or neuroprotective effects^1^,^2^. After mechanical injury, the transition from phenotype M1 to M2 ensures that the damage can be efficiently repaired^3^. In neurodegenerative diseases, however, the balance between M1 and M2 microglial activation is broken down, but the switch in phenotypes depends on the disease stages and severity^2^. Microglial responsiveness to injury can thus serve as a diagnostic markers of disease onset or progression^4^.

In neurodegenerative diseases, microglia proliferate and adopt an activated state - a process referred to as priming - which renders microglia susceptible to a secondary inflammatory stimulus, and is thought to exacerbate disease progression^4^. Evidence for involvement of microglia in neurodegenerative diseases is supported by the post mortem assessment of human brain tissue, animal models, and by positron emission tomography (PET) studies with radiolabeled translocator protein (TSPO) ligands in patients^5^. Crucially, a number of genetic risk factors in Alzheimer’s disease (AD) were found to be linked to microglial function, compromising phagocytic efficiency and clearance of amyloid deposits as well as enhancing neuroinflammation^6^. Acute or prolonged systemic infection or inflammation triggers neuroinflammation contributing to the onset and progression of AD^7^. Therefore, glial activation is of great interest as a potentially key modifiable pathological mechanism in AD and other neurodegenerative disorders, and as a mechanistic biomarker for the development of new treatment options targeting neuroinflammation or a dysfunctional amyloid metabolism.

In the development for novel treatments for neurodegenerative diseases, non-invasive biomarkers are essential tools to monitor experimental treatment options, or to assess which subgroups of patients at risk or even preclinical populations may benefit most from potential, pre-emptive treatments. Currently, the best characterized imaging biomarker for inflammation is based on the upregulation of the translocator protein 18 kDa (TSPO), a marker of microglia activation^8^. Increased expression of TSPO by activated microglia or macrophages in the central nervous system has been reported in various neuroinflammatory conditions and has been recognized as a hallmark of neuroinflammation^9^,^10^. Radiolabeled TSPO ligands for PET have been used to image neuroinflammation in animal models^11^,^12^, and in clinical studies^13^. Binding of the TSPO ligand ^11^C-PBR28 indicated that neuroinflammation correlates with the severity of cognitive decline in patients with AD^14^. However, nonspecific binding, the need for tracers with a short half-life, and the limitations on serial monitoring in at-risk patient groups imposed by the ionizing radiation load makes the search for a complementary method using magnetic resonance imaging (MRI) attractive.

While current MRI methods lack sensitivity and are not specific to inflammatory processes or microglial activation^15^, a number of metabolites that are readily quantifiable with clinical ^1^H MR spectroscopy have been proposed to indicate glial activation associated with neuroinflammatory conditions. Changes in myoinositol (mI), glutamate/glutamine, total creatine, choline containing compounds and *N*-acetylaspartate (NAA)^16^,^17^, but also lactate and lipids^18^ have been reported in response to neuroinflammation or in inflammatory diseases. Administration of lipopolysaccharide (LPS) is the most common procedure to activate microglia through stimulation of toll-like receptor 4, which dose-dependently induces secretion of inflammatory mediators causing neuroinflammation at moderate doses to severe sepsis at very high doses. When stimulating human microglial cells in culture with LPS, elevated glutamate and lactate levels were found^19^, while an increase in lactate levels and a decrease in choline were observed with *ex vivo* MRS after an acute extreme dose of LPS (32 mg/kg i.p. administration)^20^. With an intracerebral dose of 1 mg/kg LPS, increases in macromolecule levels but no difference in other metabolites were seen together with widespread astrogliosis and increased levels of pro-inflammatory cytokines in rat pups^18^. A recent MRS study saw an increase in NAA, GABA and tCr after 500 μg/kg LPS i.p., but no change after 50 μg/kg i.p.^21^, yet how this relates to glial activation was not assessed. Overall, there is limited knowledge as to which metabolite(s) would best relate to microglial activation or neuroinflammation, partly due to the different experimental conditions.

In neurodegenerative diseases, microglial function is compromised and an altered responsiveness to immune challenges can therefore be expected^4^, contributing to the detrimental effect of systemic infection on disease progression^7^. Low systemic doses of LPS (e.g. 100–500 μg/kg) can reveal dysfunctional microglia responses to systemic infection as they induce more detrimental effects in preclinical models of neurodegenerative diseases than in control animals^22^. During the acute phase of the LPS response, morphological activation of microglia appears within four hours in the primed brain^23^. We were therefore particularly interested to establish whether MRS can discriminate the acute microglial response to LPS between an APP/PS1 mouse model of AD and wild-type (WT) mice. To address this aim, we used a dose of 100 μg/kg i.v., which was found to induce a heightened sickness syndrome, cognitive impairments and inflammatory response in the primed brain compared with control animals^24^,^25^. We focused on the hippocampus, which is one of the earliest brain region affected in AD, contributing to cognitive decline^26^,^27^.

We hypothesised that the microglial response to LPS will be greater in APP/PS1 mice than in WT mice, and that this will be reflected by a distinct metabolic response as revealed by *in vivo* MRS. In particular, we expected that levels of mI will increase in response to LPS in animals with a pre-existing chronic brain disorder thought to compromise microglial function, compared with controls. Myoinositol is considered to be a marker of glial cells sensitive to anti-inflammatory treatments, based on a comprehensive series of NMR studies in cell culture^28^,^29^.

Our findings show that after an acute challenge with a low dose of LPS, a microglial phenotype consistent with higher activation levels was correlated with higher macromolecule and lipid levels in WT mice. In contrast, APP/PS1 mice showed higher mI levels, but no microgliosis.

## Methods

### Ethical statement

All procedures were authorised and approved by the University of Nottingham ethics committee and UK home office under project license 40/3601, according to the UK Animals (Scientific Procedures) Act 1986. Data are reported according to the ARRIVE guidelines for *in vivo* experiments^30^.

### Animals

APPswe/PS1dE9 (APP/PS1) transgenic mice on a C57BL6/J background and WT C57BL6/J littermates mice were bred in the University of Nottingham’s Biomedical Service Unit as previously described^31^ or purchased from Charles River UK. Genotyping was performed by Transnetyx (Cordova, TN, USA), and confirmed by immunostaining of β-amyloid (Aβ) plaques (data not shown). 36 experimentβlly naïve male mice were used, but one poorly responded to prolonged anesthesia and was humanely euthanized. This resulted in 17 APP/PS1 mice (mean age: 230 days, range 128–341 days; mean *ad libitum* weight: 36 g; range: 34–38 g) and 18 age-matched WT mice (mean age: 233 days, range 127–346 days; mean *ad libitum* weight: 35 g; range: 33–37 g). The age range covered early (4.5 months of age) and moderate (8–11 months) stages of pathology and was controlled with aged matched animals in each experimental group (Suppl. Fig. 1). Mice were maintained in individually vented cages under standard husbandry conditions on a 12/12 h light cycle, with lights on at 07:00 h; the room temperature, relative humidity and air exchange were automatically controlled. Animals were group-housed (usually three per cage unless they showed sign of aggression), with *ad libitum* access to food and water, and provided with nesting material and a play tube.

### Drug treatment

Lipopolysaccharide (LPS, Escherichia coli serotype, Sigma0111:B4, Sigma Aldrich) was dissolved in phosphate buffer saline (PBS, Sigma Aldrich) at a concentration of 200 μg/ml, and stored in aliquots at −20° until use. The day of the experiment LPS was further diluted 1:2.5 in PBS to a final concentration of 80 μg/ml. Mice were injected intravenously (i.v.) in the lateral tail vein with 100 μg/kg LPS or an equivalent volume of PBS, at a flow rate of 25 μl/min using a 1.0 cc syringe connected to a tail vein catheter made of a 27G ½” gauge needle and polyethylene PU tubing.

### Study design

**Figure 1:**
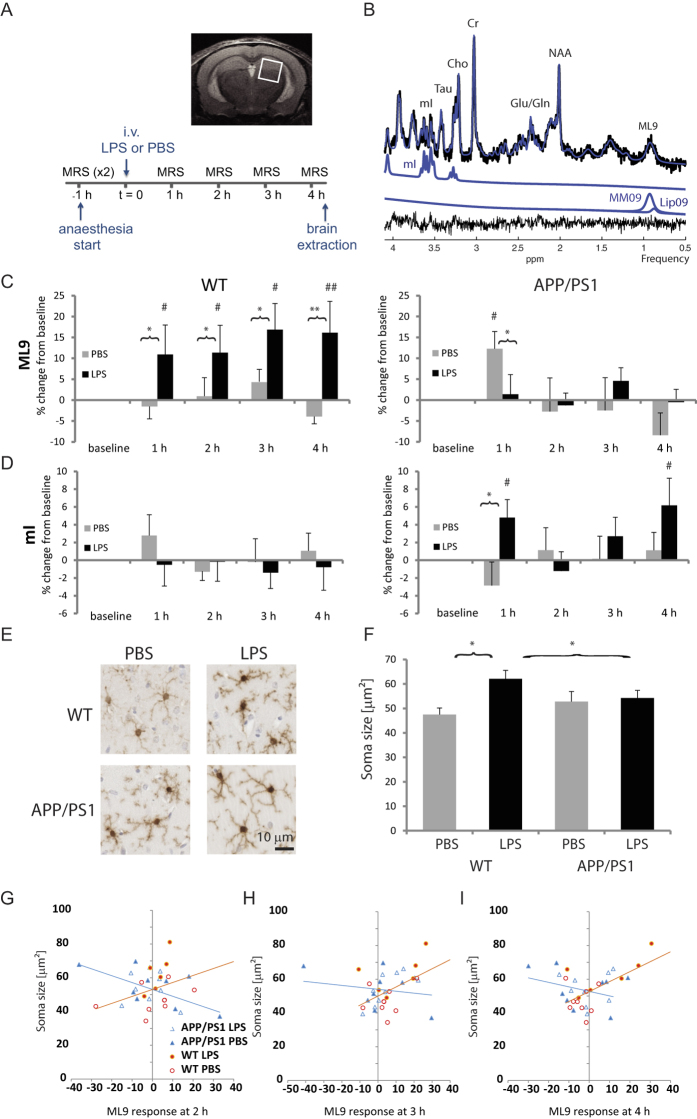
APP/PS1 and WT mice respond differentially to LPS. (**A**) Timeline of the experiment measuring the hippocampal metabolic response to LPS; inset: voxel placement. WT and APP/PS1 mice were subjected to two baseline MRS scans in a region of interest covering the hippocampus prior to receiving a tail vein injection of lipopolysaccharide (LPS, 100 μg/kg) or its vehicle (phosphate buffer saline, PBS). The metabolic response to the intervention was monitored for 4 hours. (**B**) Representative MRS spectrum (top), the fitted spectrum (blue), the residuals (bottom, black) and individual metabolite fitted spectra for myoinositol (mI) and macromolecules and lipids at 0.9 ppm (ML9). (**C**) Mean ± SEM percent changes in ML9 levels relative to sum of selected metabolites. In WT mice, LPS induced a sustained increase in ML9 levels which was not observed in APP/PS1 mice. (**D**) Mean ± SEM percent changes in mI levels relative to sum of selected metabolites. In APP/PS1 mice, LPS significantly increased mI levels at 1 and 4 hours post injection. (**E**) Representative Iba1 positive microglia (40× magnification) shown for WT (top row) and APP/PS1 (bottom row) mice, 4 hours after injection of either PBS (left) or LPS (right). (**F**) Mean ± SEM soma size. Microglia activation, reflected by an increase in soma size, occurred in response to LPS in WT but not APP/PS1mice. Changes in soma size were (**G**) negatively correlated to ML9 in APP/PS1 mice at 2 hours (r = −0.575, p = 0.02), but positively correlated to ML9 in WT at (**H**) 3 h (r = 0.514, p = 0.035) and (**I**) 4 h (r = 0.64, p = 0.005) suggesting that microglia activation is associated with increasing ML9. *p < 0.05, **p < 0.01 compared with PBS; ^#^p < 0.05, ^##^p < 0.01 compared with baseline levels.

APP/PS1 transgenic mice and age-matched WT controls were randomly assigned to each treatment condition: LPS (n = 9 per genotype) or PBS (8 APP/PS1 and 9 WT) as control for the effect of prolonged anesthesia, which may differentially affects AD mouse models and WT mice^32^. Experiments were performed in blind (genotype and treatment). Animals were anesthetized with a mixture of oxygen and isoflurane (Isocare, 3% for induction and 1–2% for maintenance), and positioned in the imaging system in a custom made frame to minimize movement. Eye gel (Lubrithal) was applied in both eyes to avoid desiccation. Body temperature and breathing rate (90–120 breaths/min) were monitored and maintained stable throughout the experiment. The timeline of MRS experiments is shown in Fig. 1A. Two MRS scans were acquired to establish a baseline of brain metabolite levels. Each animal then received either LPS or PBS, delivered through an i.v. line while remaining positioned in the MRI system. To track the metabolic response, an MRS spectrum was then acquired every 60 min for four hours. Brains were then immediately extracted and processed for histology.

### MRS acquisition

MR recordings were performed on a horizontal 7 T system (Bruker, Karlsruhe, Germany), bore size 30 cm, with ParaVision 5.1 software. A 72 mm volume coil was used for excitation and a quadrature surface coil for signal detection (Bruker). Anatomical scans were acquired using a RARE sequence (Rapid Acquisition with Relaxation Enhancement)^33^ in coronal, sagittal and axial orientation (RARE factor 8, TR = 5 s, TE = 11.8 ms, matrix size 256 × 256, field of view, FOV 15 × 20 cm, 30slices, slice thickness 0.5 mm). For MR spectroscopy, a single voxel of 2 × 2 × 2 mm^3^ was centered on the right hippocampus (Fig. 1A). To optimize field homogeneity, shims were first adjusted with a global field map based shim (MAPSHIM, ParaVision 5.1, Bruker; Germany) followed by a local shim (FASTMAP)^34^. *In vivo* MR spectroscopy scans were acquired with a Point-Resolved Spin echo Sequence (PRESS)^35^ with a total acquisition time of 22 min (TR/TE = 2500/13 ms, 512 averages, 8 dummy scans, 2048 acquisition data points, spectral width 4006 Hz) with VAPOR (variable power and optimized relaxation delays)^36^ water suppression (bandwidth 200 Hz). A reference scan without water suppression was acquired for frequency and eddy current correction during each acquisition cycle.

### MRS data analysis

A representative ^1^H MR spectrum from a voxel positioned unilaterally across the hippocampus and the upper part of the thalamus is shown in Fig. 1B. Across all spectra, the average metabolite line width (full width at half maximum) was 9.9 ± 0.2 Hz, and the signal to noise ratio was 8.2 ± 0.2, as reported by LCModel. Data were analysed using LCModel for estimation of metabolite concentrations^37^. The analysis window chosen was 0.5 ppm to 4.1 ppm; data were pre-processed with zero-order phasing, referencing and residual water line removal. Data were fitted to a linear combination of 17 metabolites in a simulated basis set containing: alanine, aspartate, creatine, phosphocreatine, γ-aminobutyric acid, glucose, glutamine, glutamate, glycerophosphorylcholine, phosphorylcholine, glutathione, mI, lactate, NAA, n-acetyl aspartatyl glutamate, *scyllo*-inositol and taurine. Spectra were considered if the linewidth reported by LCModel did not exceed 20 Hz. Metabolite concentrations derived from fitted spectra consistently within Cramér-Rao bounds <15% were included in further analysis performed with custom-made software (Matlab, The Mathworks, Nattick, MA). We determined a neurochemical profile of total NAA (tNAA), total choline (tCho), total creatine (tCr), mI, taurine, glutamate (Glu), and the combined signals of glutamate and glutamine (Glx), and of lipids and macromolecules at 0.9 ppm (ML9). Baseline values prior to LPS administration were calculated as the average of the two baseline scans.

Differences in creatine levels have been reported between transgenic models of AD and controls^38^,^39^. Relative metabolite concentrations were therefore expressed as the ratio to the sum of metabolites mI, tNAA, tCho, tCr and Glx to reduce the impact of possible variations in the creatine peak^40^. This method also controls for changes in creatine levels, which can be expected in AD^41^.

### Immunohistochemistry

Brains were post-fixed in 4% paraformaldehyde, stored at 4–8 °C for a minimum of 48 hours, and then embedded in paraffin wax on a tissue embedding station (Leica TP1020). 7 μm-thick coronal sections were cut throughout the hippocampus using a microtome (Microtome Slee Cut 4060), mounted on APES coated slides and dried overnight at 40 °C. Immunostaining of the microglial marker Iba1 (ionized calcium binding adaptor molecule 1) and the astrocyte marker GFAP (glial fibrillary acidic protein) was carried out using standard protocols. Sections were first re-hydrated in consecutive rinses in xylene, 100% ethanol, 70% ethanol and dH_2_O and then immersed in sodium citrate buffer for 20 minutes at 95–99 °C for antigen retrieval. Once the solution was cooled down to 70 °C, sections were washed in PBS, incubated in 1% H_2_O_2_ solution to inhibit endogenous peroxidase activity, washed in PBS and blocked in 5% goat serum. Brain slices were then incubated with rabbit anti-Iba1 (Wako, cat. nr. 019–19741; 1:6000 in PBS-T) or anti-GFAP (Biogenix, cat. nr. AM020-5M, 1:4000 in PBS-T) antibody for 1 h at room temperature, washed in PBS, and incubated with biotinylated secondary antibody (Vectastain Elite ABC Kit, Rabbit IgG, Vector Labs, Burlingame, CA cat. nr. PK-6101, 1:200 in PBS-T) for 30 min. Tissue was washed, exposed to ABC-HRP (Vectastain Elite ABC Kit R.T.U, Vector Labs, cat. nr. PK-7100) and labelled with DAB peroxidase substrate (Vector Labs cat. SK-4100) according to manufacturer’s instructions. Brain slices were then counterstained using a haematoxylin and eosin protocol, dehydrated in increasing concentrations of alcohol and xylene for 2 min, and mounted using Clearvue mountant (Thermo Scientific, cat. nr. 4212). Digital focused photo-scanning images were then acquired using a Hamamatsu NanoZoomer-XR 2.0-RS C10730 digital scanning system with TDI camera technology a NanoZoomer (Hamamatsu Photonics K.K. Systems, Japan) at 20× magnification and visualised using NDP.view2 (NanoZoomer Digital Photography).

### Semi-automated analysis of Iba1 and GFAP immunostaining

For extraction of morphometric features, a region of interest (ROI) was drawn on the digitized histology images at 20× magnification using custom made software (Matlab), outlining the region covered by the MRS spectroscopy voxel (2 × 2 mm^2^, extending over the hippocampus and thalamus). For feature recognition (using custom made software programmed in Matlab), soma were first identified in the ROIs on histological images by blurring with an average filter of adjacent pixels and thresholding adapted for uneven background staining. All images were inspected and corrected manually to avoid artifacts. This provided the area occupied by glial cells and isolated microglial processes as a percentage of the region of interest, also providing the number of cells, the position of the soma and the soma size. Illustrative examples of extracted immunopositive Iba1 and GFAP cells are shown in Suppl. Fig. 2. We stained four brain slices per animal, but generally used three in the analysis.

### Experimental outcomes

The percentage area stained by Iba1 and the number of Iba1 positive cells within the ROI were used as markers of microglial density, and the soma size as a morphometric marker of microglia activation known to be sensitive to LPS^42^,^43^. The main advantage of using soma size as a marker is that it represents an objective continuous measure, sensitive to the degree of activation, and is therefore more suited to correlation analysis than other morphometric, categorical measures that rely on estimating morphological properties of microglia. For GFAP immunostaining, the percentage of stained area was used. Primary outcome measures from MRS were the normalized metabolite concentrations of mI, while secondary measures were Glx, tCr, tCho, tNAA, as well as macromolecules and lipids.

### Data analysis

Data are presented as mean ± SEM (standard error of the mean) and were analysed using InVivoStat^44^. The effect of age was first tested on baseline metabolite levels but was not found to be a significant. Age was also not a significant covariate for time course data and histological measures despite the progressive nature of amyloid deposition, therefore all mice were regrouped by genotype. Possible genotype differences in baseline metabolite concentrations were analyzed using two-way ANOVAs. For each metabolite, the response to LPS was expressed as percent change from baseline, pre-injection levels, where the levels measured in the two baseline scans were averaged. To assess the genotype-dependent time course of metabolic responses to LPS vs. control PBS, two-way ANOVAs with genotype (WT vs. APP/PS1) and drug (PBS vs. LPS) as between subject factors and repeated measure over time were used with baseline values as a covariate. Because in transgenic mouse models of AD, microglia with the morphological appearance of an activated phenotype cluster around amyloid plaques^45^, glial markers were analysed using two-way ANOVAs with genotype and drug as between subjects factors, and the number of microglia clusters as a covariate. ANOVAs were followed by post-hoc planned comparisons, when appropriate, as relevant comparisons were planned in advance and the study designed accordingly, thereby reducing the risk of false positives. Post-hoc analyses tested whether metabolic responses to LPS or PBS were significantly above baseline levels within each experimental group, and compared the effect of LPS vs. PBS at each time point within each genotype. Relationships between glial and metabolic responses, and whether the associations were dependent upon the genotype, were tested using linear regression models with an interaction term between genotype and metabolite. Given any significant genotype effect, relevant associations between glial and metabolic responses to LPS were then calculated for each genotype using the Pearson correlation coefficient. P ≤ 0.05 was considered statistically significant.

## Results

There was no age-dependent effect of the genotype on baseline pre-injection metabolite levels (Suppl. Fig. 1). Levels did not differ between APP/PS1and WT mice, except for tCr levels which were slightly but significantly lower in APP/PS1 mice (p = 0.0025 compared to WT, Table 1). The main statistical effects in the time course of metabolic response to LPS and PBS are reported in Suppl. Table 1, and metabolite concentrations are shown in Suppl. Table 2.

### Differences between WT and APP/PS1 in response to vehicle injection

**Figure 2:**
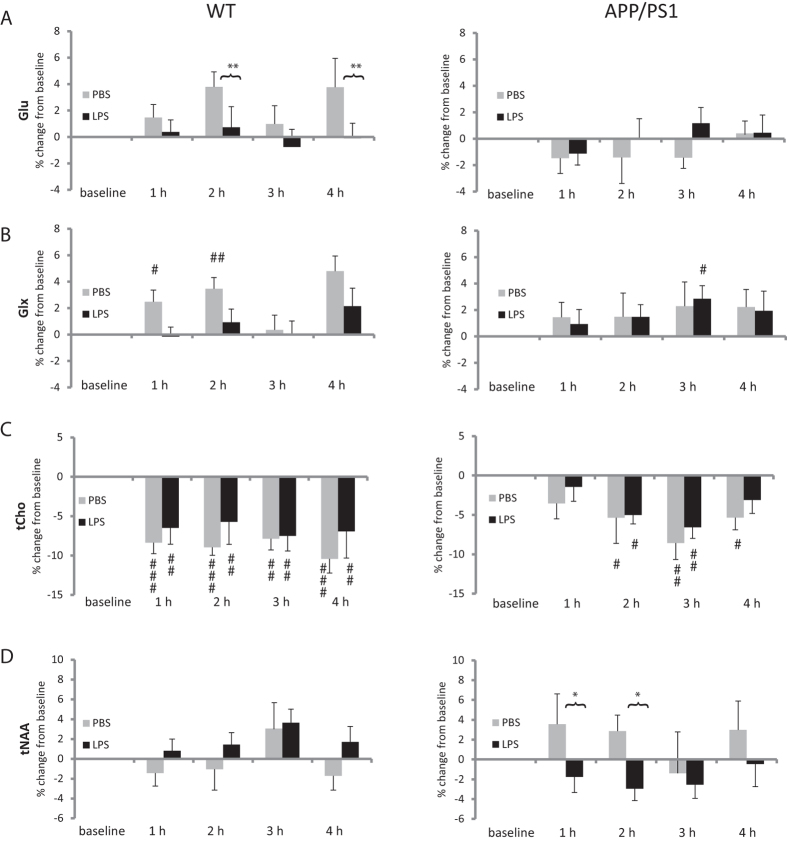
Metabolic response to anaesthesia and vehicle administration. Data are presented as mean ± SEM percent changes in metabolites levels relative to baseline. Metabolites levels were calculated as ratio of the sum of selected metabolites. LPS prevented the rise in (**A**) glutamate (Glu) and (**B**) glutamate + glutamine (Glx) levels occurring with time in PBS-treated WT mice. (**C**) The reduction in tCho seen with time was more pronounced in WT than APP/PS1 mice. (**D**) tNAA levels differed between LPS- and PBS-treated APP/PS1 mice the first 2 hours post-injection although they did not did not fluctuate significantly in response to either treatment. *p < 0.05 compared with PBS; ^#^p < 0.05, ^##^p < 0.01, ^###^p < 0.01 compared with baseline levels.

**Figure 3:**
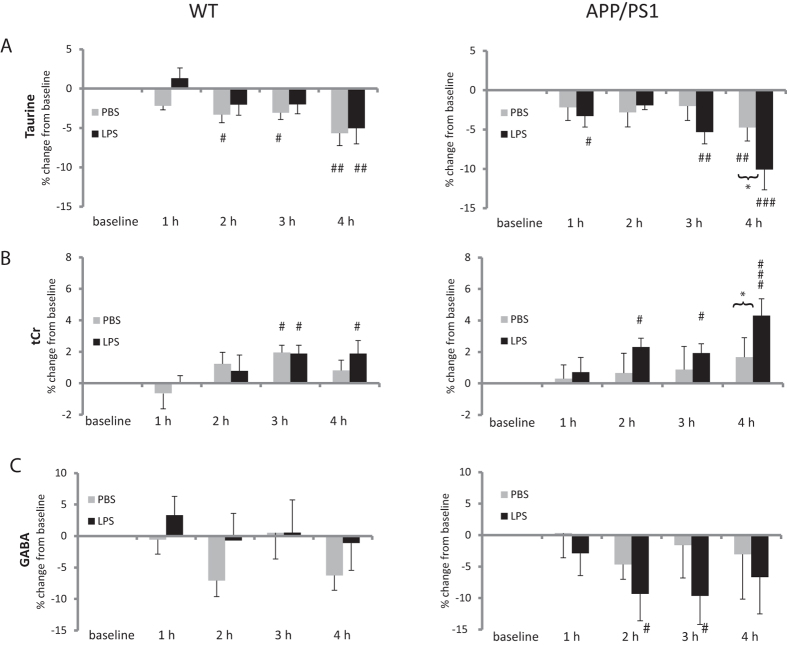
Late metabolic response in APP/PS1 mice. Data are presented as mean ± SEM percent changes in metabolites levels relative to baseline. Metabolites levels were calculated as ratio to the sum of selected metabolites. (**A**) Taurine levels decreased with time regardless of genotype and treatment, suggesting a response to prolonged anaesthesia. In APP/PS1 mice, however, the reduction in taurine levels was significantly exacerbated by LPS at 4 h. (**B**) Opposite effects were seen for tCr levels, which increased with time, an effect exacerbated by LPS at 4 h in APP/PS1 mice. *p < 0.05, **p < 0.01 compared with PBS; ^#^p < 0.05, ^##^p < 0.01, ^###^p < 0.01 compared with baseline levels.

Injection of PBS, used as control for the experimental set-up and prolonged anaesthesia, was associated with time-dependent changes in the levels of some metabolites. These were more pronounced in WT than APP/PS1 mice. WT mice showed increased Glu levels at 2 and 4 hours (both p = 0.006 vs. baseline, Fig. 2A), and increase Glx at 1, 2 and 4 hours (vs. baseline, p = 0.02; p = 0.002 and p < 0.0001, respectively, Fig. 2B). Taurine levels decreased in both WT mice (vs. baseline: t = 2 h, p = 0.035; t = 3 h, p = 0.049, t = 4 h, p = 0.0004, Fig. 3A) and APP/PS1 mice (vs. baseline: t = 4 h, p = 0.008, Fig. 3A). tCho levels showed a greater decrease in WT (vs. baseline: t = 1 h, p = 0.0026; t = 2 h, p = 0.0076; t = 3 h, p = 0.0005, t = 4 h, p = 0.0013, Fig. 2C) than in APP/PS1 mice (vs. baseline: t = 2 h, p = 0.026; t = 3 h, p = 0.0005, t = 4 h, p = 0.027, Fig. 2C). There was no anaesthesia or vehicle effect in ML9 or mI.

### WT and APP/PS1 animals show contrasted metabolic responses to LPS

WT mice responded to LPS exposure with a sustained increase in the combined macromolecule and lipid signal at 0.9 ppm (ML9) over the four hours post injection (vs. baseline: t = 1 h, p = 0.014; t = 2 h, p = 0.012; t = 3 h, p = 0.0002, t = 4 h, p = 0.0004, Fig. 1C), which was not seen in APP/PS1 mice although LPS prevented the increase in ML9 seen in PBS-treated APP/PS1 mice at 1 hour (p = 0.029, Fig. 1C). In WT mice, LPS also prevented the rise in Glu seen at 2 and 4 hours post injection (vs. PBS-treated WT mice: t = 2 h, p = 0.01; t = 4 h, p = 0.003; Fig. 2A) and in Glx at 4 hours only (vs. PBS-treated WT mice: t = 4 h, p = 0.05; Fig. 2B).

The response of APP/PS1 mice to LPS was characterised by changes in mI, taurine, and tCr not seen in WT animals. Myoinositol levels of APP/PS1 mice were significantly higher than at baseline levels at 1 and 4 hours after LPS administration (p = 0.037 and p = 0.008, respectively, Fig. 1D). With time, taurine levels decreased while tCr levels increased regardless of genotype, but in APP/PS1 mice, LPS selectively exacerbated these changes at the last time point (p = 0.007 for taurine, Fig. 3A. and p = 0.023 for tCr, Fig. 3B, compared to PBS-treated APP/PS1 mice).

LPS had no impact on tCho levels, which were significantly decreased throughout the experiment to a greater extent in WT than APP/PS1 mice (Fig. 2C) or tNAA which did not fluctuate significantly during the course of the experiment (Fig. 2D).

### APP/PS1 mice fail to show a microglial response to LPS

**Figure 4:**
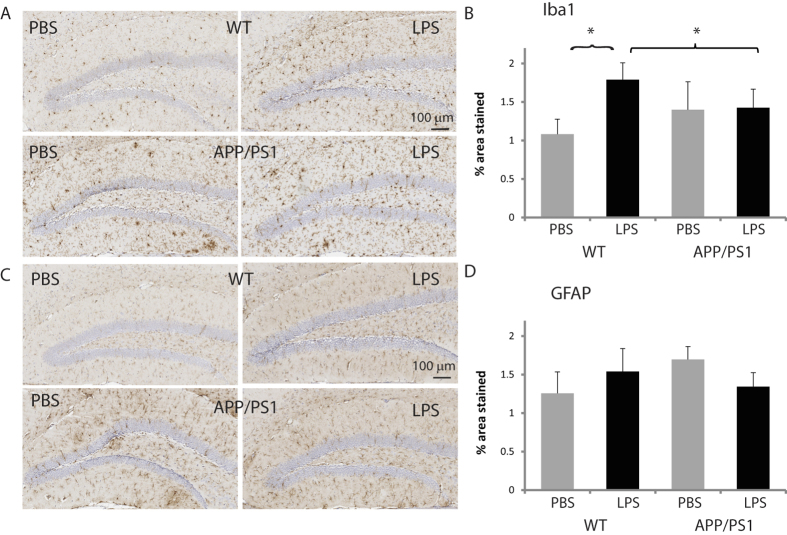
Lack of reactive microgliosis in APP/PS1 mice. (**A**) Representative Iba1 positive microglia (10× magnification) shown for WT (top row) and APP/PS1 (bottom row) mice, 4 hours after injecting either PBS (left) LPS (right). (**B**) Microglial density (mean ± SEM area stained by Iba1) was increased by LPS in WT but not in APP/PS1 mice. (**C**) Representative GFAP positive astrocytes (10× magnification) shown for WT (top row) and APP/PS1 (bottom row) mice, 4 hours after injecting either PBS (left) or LPS (right). (**D**) Mean ± SEM area stained by GFAP. Astrogliosis was not observed in response to LPS. *p < 0.05.

In WT mice, LPS significantly increased the percentage area stained by Iba1 by about 65% (p = 0.032 compared to PBS-treated WT mice, Fig. 4A,B) and microglia soma size by about 31% (p = 0.0007 compared to PBS-treated WT mice, Fig. 1E,F), suggesting that these immunoreactive cells were activated. In contrast, LPS did not induce microgliosis in APP/PS1 mice. The number of microglial cells (not shown) and the % area stained by GFAP (Fig. 4C,D), were not altered by LPS.

### Associations between metabolic and glial responses to LPS

To investigate whether changes in specific metabolites predict microglial responses to LPS in WT and APP/PS1 mice, association studies were performed. The major finding is that the rate of changes in ML9 levels differentially predict microglia soma size in WT and APP/PS1 mice at 2 (p = 0.01), 3 (p = 0.036) and 4 (p = 0.006) hours post injection. Changes in ML9 levels were positively correlated with microglia soma size in WT mice (t = 2 h, r = 0.378, p = 0.13, Fig. 1G; t = 3 h, r = 0.514, p = 0.035, Fig. 1H, t = 4 h, r = 0.64, p = 0.005, Fig. 1I), but negatively correlated in APP/PS1 mice (t = 2 h, r = −575, p = 0.02, Fig. 1G ; t = 3 h, r = −0.167, p = 0.54, Fig. 1H, t = 4 h, r = −0.302, p = 0.25, Fig. 1I). Therefore, a greater soma response to LPS was associated with a greater increase in ML9 levels in WT mice but the opposite is expected in APP/PS1 mice.

## Discussion

We followed the metabolic response to a mild immune challenge in a mouse model of AD and healthy controls and related the response to histological outcome measures that reflect microglial and astrocyte activation. In WT mice, LPS induced a microglial phenotype consistent with activation, associated with a correlated increased in macromolecule and lipid levels at 0.9 ppm (ML9). In APP/PS1 mice, in contrast, LPS did not affect the morphological glial markers, but increased mI levels at one and four hours post-injection, while also inducing late changes in taurine and tCr levels.

Metabolic changes have been reported in the hippocampus of a number of mouse models of AD, some of which were found to be age-dependent^46^,^47^,^48^,^49^. There are, however, discrepancies between studies, which can be attributed to differences in mouse model or study design. We used APPswe/PS1dE9 mice on a C57BL/6 background at early (4.5 months) and moderate (8–11 months) stages of amyloid plaque deposition, but baseline pre-injection metabolite levels did not differ significantly as a function of age in either genotype. Consistent with our findings, APPswe/PS1dE9 mice backcrossed on a C57BL6 background did not differ from their wild-type littermates at 8 months of age for the same metabolites (tCho, tCr, NAA, Ins, Tau, Glu, Glx), although NAA was reduced in the AD mouse model when the scan was repeated at 12 months of age^49^. In contrast, increased levels of mI were found at 3, 5 and 8 months of age in APPswe/PS1dE9 mice on a mixed genetic background compared to their WT littermate while glutamate and NAA were reduced at the two later time points^48^. These data are in apparent contradiction to ours, but differences in the genetic background can contribute significantly to phenotype variability^50^. Furthermore their longitudinal study lacked controls for the effect of repeated anaesthesia. Although this was not tested systematically, repeated anaesthesia appeared to exacerbate age-related changes in some metabolites, particularly mI, in an APP/PS1 model^46^. However, changes in mI are not consistent, as another study did not report changes in mI levels at three months of age in APPswe/PS1dE9 mice on the same mixed genetic background^51^. Furthermore, while a reduction in NAA levels appear to be the most consistent change seen in APP/PS1 mice, in our age range, they are unlikely observed in mice naïve to the anaesthesia.

The metabolic profile of WT and APP/PS1 mice did not differ at baseline, but their metabolic response to an immune challenge was contrasted independently of their ages. Low LPS doses have allowed to discriminate differential susceptibility to immune challenges between animals models of neurodegenerative diseases and healthy controls^22^. Accordingly, we found that LPS induced microglial activation - at 100 μg/kg - was associated with a sustained increase in ML9 levels in WT mice, but these responses were not seen in APP/PS1 mice. An increase in macromolecules at 0.9 ppm trending towards significance has been reported previously in response to a pro-inflammatory 1 mg/kg LPS dose in developing rats^18^. This signal is generally attributed a broad spectral peak of macromolecules and lipids around 0.9 ppm^52^, but its origins and biological significance are not well defined. Macromolecules comprise large molecules, including proteins, lipids and nucleic acids. Combining high resolution NMR and HPLC (high pressure liquid chromatography), Kauppinen *et al.*^53^,^54^ investigated the polypeptide signals at 0.9, 1.22 and 1.4 ppm, and were able to determine that the peak could be attributed mostly to proteins, and more specifically to thymosin β4, a peptide expressed in microglia^55^. The glial response to immune challenges involves a balance between M1 and M2 phenotypes, and the release of pro- and anti-inflammatory molecules^2^,^3^. Thymosin β4 has been proposed as a neuroprotective and anti-inflammatory agent^56^,^57^. Thus, given our observation of a positive association between ML9 and LPS-induced soma enlargement, ML9 levels could be a marker of microglial response to an immune stimulus, but whether it relates to the protective M2 phenotype of microglia has to be confirmed in future work.

The lack of ML9 response in APP/PS1 mice is consistent with the lack of microglial response to LPS, but intriguing. Mouse models of AD are indeed expected to be more susceptible to pro-inflammatory challenges because of underlying microglial priming^4^. However, in the age range we investigated, microglia density and soma size were not significantly increased in APP/PS1 compared to WT mice in the PBS-treated groups or after the immune challenge. This partly explains the lack of morphological activation of microglia after LPS in our APP/PS1 as it appears within four hours in the primed hippocampus^23^ and within 4–8 hours in the healthy brain^58^. However, the compromised immune cells in AD could also result in a state of reduced response to peripheral infections^59^, a hypothesis more consistent with our observation of the development of microgliosis at 4 hours post LPS in WT but not in APP/PS1 mice. ML9 levels and soma size correlated negatively in APP/PS1 mice suggesting that the larger the microglia, the less they respond to LPS. This is in agreement with reports that microglia - clustering around amyloid plaques for Aβ clearance- show an apparent activated morphology, despite being dysfunctional^45^. Microglial phenotypes are complex in AD. While M1 microglia appear to be impaired in their ability to remove Aβ, M2 microglia have been demonstrated to be efficient phagocytes^3^ and the presence of Aβ plaques leads to an uneven distribution between M1 and M2 microglia^2^. Microglia surrounding the plaques for Aβ phagocytosis generally manifest an M2 activation phenotype, but their inability to react properly promotes Aβ accumulation, and proinflammatory stimuli such as LPS limit their phagocytotic potential^3^. Thus, this also supports the hypothesis that ML9 may relate to the M2 phenotype of microglia.

In APP/PS1 mice, the lack of microglial and ML9 responses to LPS was associated with changes in mI, taurine and tCr levels at four hours post injection. Myoinositol is considered to be a marker of glial cells sensitive to inflammation^28^,^29^. In a model of multiple sclerosis, increased mI levels were observed in areas of increased gliosis^60^. However, more recent studies suggest that mI levels in AD reflect amyloid plaque load rather than glial cells activation. Shiino *et al.* found mI levels elevated in AD, but not in ischaemic vascular dementia^61^. Murray *et al.* compared metabolite levels seen in *ante mortem* MRS with post mortem histology in AD patients of different stages of disease progression^17^. The investigators found that elevated mI levels and reduced NAA/mI were associated with higher amyloid-beta plaque load, but not with increased in GFAP-positive astrocytes (indicating changes in astrocyte proliferation) and CD68-positive microglia (indicating neuroinflammation) in a patient cohort with more advanced AD. They concluded that there was no relationship between mI elevation and gliosis in AD. This conclusion is supported by the observation that, while WT mice in our study showed a microglial phenotype consistent with activation, this microglial phenotype was not associated with elevated mI levels. A more recent study investigating passive immunization with amyloid-beta antibodies in the APP/PS1 mouse model found an attenuation of the increased mI in the treated mice compared with placebo^62^. Microglia are known to have a role in the clearance of amyloid deposits. Amyloid-beta antibodies and immunization were found to clear plaque load and activate microglia at the same time suggesting that they stimulate their phagocytic activity^63^,^64^. This again supports the association of mI with amyloid-beta.

It is possible that the late increase in mI seen in APP/PS1 mice treated with LPS reflect an effect of the immune challenge on amyloid metabolism. LPS was reported to dose-dependently modulate amyloid plaque load. In APP/PS1 mice, intracerebral administration of LPS (4–10 μg) was found to rapidly clear amyloid beta deposits, showing visible effects within 3 days post LPS. This effect requires microglial activation and stimulation of its phagocytic phenotype^65^,^66^,^67^. In contrast, high systemic doses (5 mg/kg, able to induce severe sepsis) increased amyloid plaque load^68^. There is no published report of the effects of the 100 μg/kg dose on amyloid deposition, but we have observed a significant increase in amyloid plaque load in female APP/PS1 mice 7 days after a single challenge with 100 μg/kg LPS without concomitant microglial activation (unpublished data). The changes we observe in the current study in tCr and taurine have also been found to be related to amyloid deposition^38^,^69^. Moreover, repetitive anesthesia with isofluorane was found to exacerbate amyloid pathology in mouse models of AD^32^. Thus, it is possible that the metabolic responses of APP/PS1 mice to LPS in this study reflect early changes in amyloid metabolism, with the added effect of prolonged isofluorane anaesthesia. LPS lowers arterial pressure, which could have an effect on the brain metabolite concentration measured by MRS^70^.

Contrary to our initial expectations, we did not observe the increased mI response in the APP/PS1 animals to be associated with microglial activation. The response to LPS in WT mice, an increase in ML9, may reflect the glial response to a pro-inflammatory stimulus, while the metabolic response of APP/PS1 mice may be due to changes in amyloid metabolism. A limitation of the present study, however, is that early markers of the amyloidogenic pathway were not assessed. Changes in amyloid plaque load are a late event in amyloid metabolism, extending beyond the 4-hour post-injection period of this experiment. Although new plaques appear within 24 hours^71^, they mature within weeks with significant changes in size seen after 7 days^72^.

A further constraint of the study is the potentially confounding impact of prolonged anaesthesia on some metabolites, which can also be observed with relatively short exposure^73^. Anaesthesia is inevitable in preclinical imaging, and an essential methodical factor to control extreme stress.The use of isofluorane allows accurate control of the depth of anaesthesia, but AD mouse models were found to be more susceptible to anaesthesia with isofluorane than WT mice, exacerbating pathology including microglial activation and amyloid deposition with repeated exposure^32^. In our study however, we have chosen a controlled experiment, with controls for mouse genotype (i.e. WT) and drug (i.e. PBS) and there was no detectable effect of anaesthesia or vehicle on ML9 or mI levels.

## Conclusion

A biomarker based on glial activation in AD has the considerable advantage of providing mechanistic information compared with more established biomarkers based on changes in hippocampal volume. We evaluated the metabolic response over time to a mild immune challenge with LPS in a model of early AD, and argued that two readily identifiable, noninvasive metabolites, ML9 and mI, distinguish microglial responsiveness to immune mediators. Their biological significance will have to be determined in relation to the multiple phenotypes of microglia to establish their potential use as markers of microglial disease status. More specifically, our findings question the current interpretation of mI as a gliosis marker calling for further work to clarify its potential role to index dysfunctional microglial activation.

## Additional Information

**How to cite this article**: Pardon, M.-C. *et al.* Magnetic Resonance Spectroscopy discriminates the response to microglial stimulation of wild type and Alzheimer's disease models. *Sci. Rep.* **6**, 19880; doi: 10.1038/srep19880 (2016).

## Supplementary Material

Supplementary Information

## Figures and Tables

**Table 1:** Mean ± SEM baseline metabolite concentrations of WT and APP/PS1 mice expressed as ratios to the sum of selected metabolites.

	WT	APP/PS1	Genotype effect
GABA	0.072 ± 0.003	0.072 ± 0.002	F(1,32) = 0.00, p = 0.99
Glu	0.219 ± 0.002	0.221 ± 0.002	F(1,32) = 0.76, p = 0.39
Glx	0.320 ± 0.002	0.322 ± 0.003	F(1,32) = 0.34, p = 0.56
mI	0.116 ± 0.003	0.111 ± 0.002	F(1,32) = 1.80, p = 0.19
ML9	0.161 ± 0.006	0.167 ± 0.008	F(1,32) = 0.41, p = 0.53
Taurine	0.192 ± 0.005	0.202 ± 0.003	F(1,32) = 3.08, p = 0.09
tCho	0.042 ± 0.0009	0.043 ± 0.0007	F(1,32) = 0.67, p = 0.42
tCr	0.196 ± 0.001	0.190 ± 0.001	F(1,32) = 10.75, **p = 0.002**
tNAA	0.160 ± 0.003	0.155 ± 0.002	F(1,32) = 1.21, p = 0.26
