# Supplementary material for: Magnetic Resonance Spectroscopy discriminates the response to microglial stimulation of wild type and Alzheimer’s disease models
Source: Sci Rep. 2016 Jan 27;6:19880. doi: 10.1038/srep19880 (PMC4728482; doi:10.1038/srep19880)
Supplement: Supplementary Information [file srep19880-s1.pdf]

Magnetic Resonance Spectroscopy discriminates the response to microglial stimulation of wild type and Alzheimer's disease models

Marie-Christine Pardon[1*], Maria Yanez Lopez[2,5], Ding Yuchun[3], Małgorzata Marjańska[4], Malcolm Prior[5], Christopher Brignell[6], Samira Parhizkar[1], Alessandra Agostini[1], Li Bai[3], Dorothee P. Auer[2,5], and Henryk M Faas[2,5*]

[1]Neuroscience, School of Life Sciences, [2]Sir Peter Mansfield Imaging Centre, School of Medicine, [3]School of Computer Sciences, [5]Medical Imaging Unit, School of Medicine, and [6]School of Mathematics, University of Nottingham, United Kingdom

[4]Center for Magnetic Resonance Research and Department of Radiology, University of Minnesota, Minneapolis, Minnesota, USA

***Corresponding Authors:**

Henryk M. Faas, PhD
Assistant Professor in Neuroimaging
Sir Peter Mansfield Imaging Centre
School of Medicine
The University of Nottingham
Room W/B 1441 B Floor West Block
Queen's Medical Centre
NG7 2UH Nottingham
United Kingdom
Tel: 0115 823 1174
Fax: 0115 823 1180
henryk.faas@nottingham.ac.uk

Marie-Christine Pardon, PhD
Assistant Professor in Translational Neuroscience
School of Life Sciences
The University of Nottingham
Room E65
Queen's Medical Centre
NG7 2UH Nottingham
United Kingdom
Tel: 0115 823 30149
marie.pardon@nottingham.ac.uk

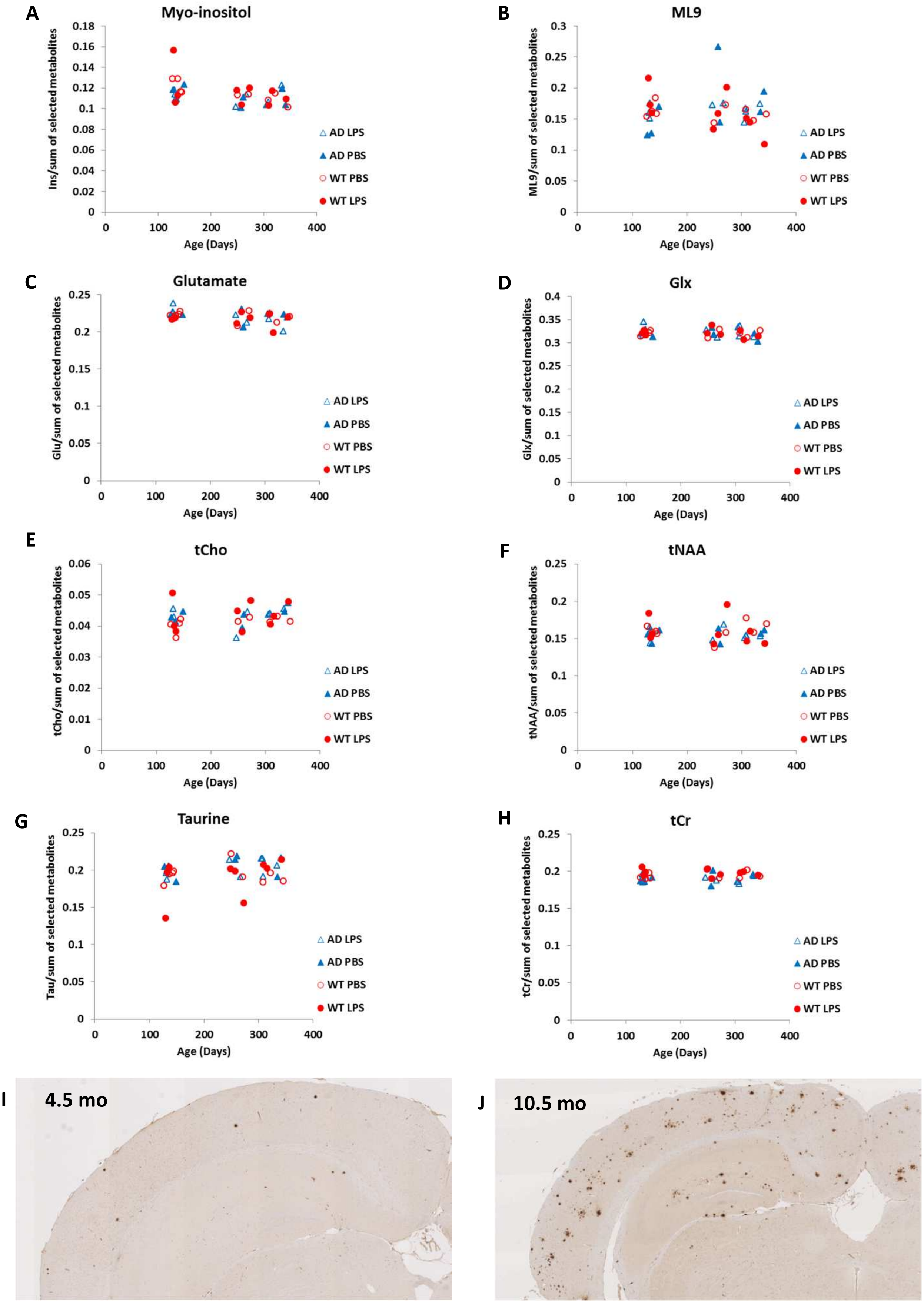

Suppl. Fig. 1: (A-H**)** Scatterplot of baseline metabolite concentrations, expressed as ratios to the sum of selected metabolites, and (I-J) representative amyloid plaque load in the hippocampus and cortex at mild (4.5 months old i.e. ~ 120 days) and moderate (10.5 months old i.e. ~320 days) stage of pathology.

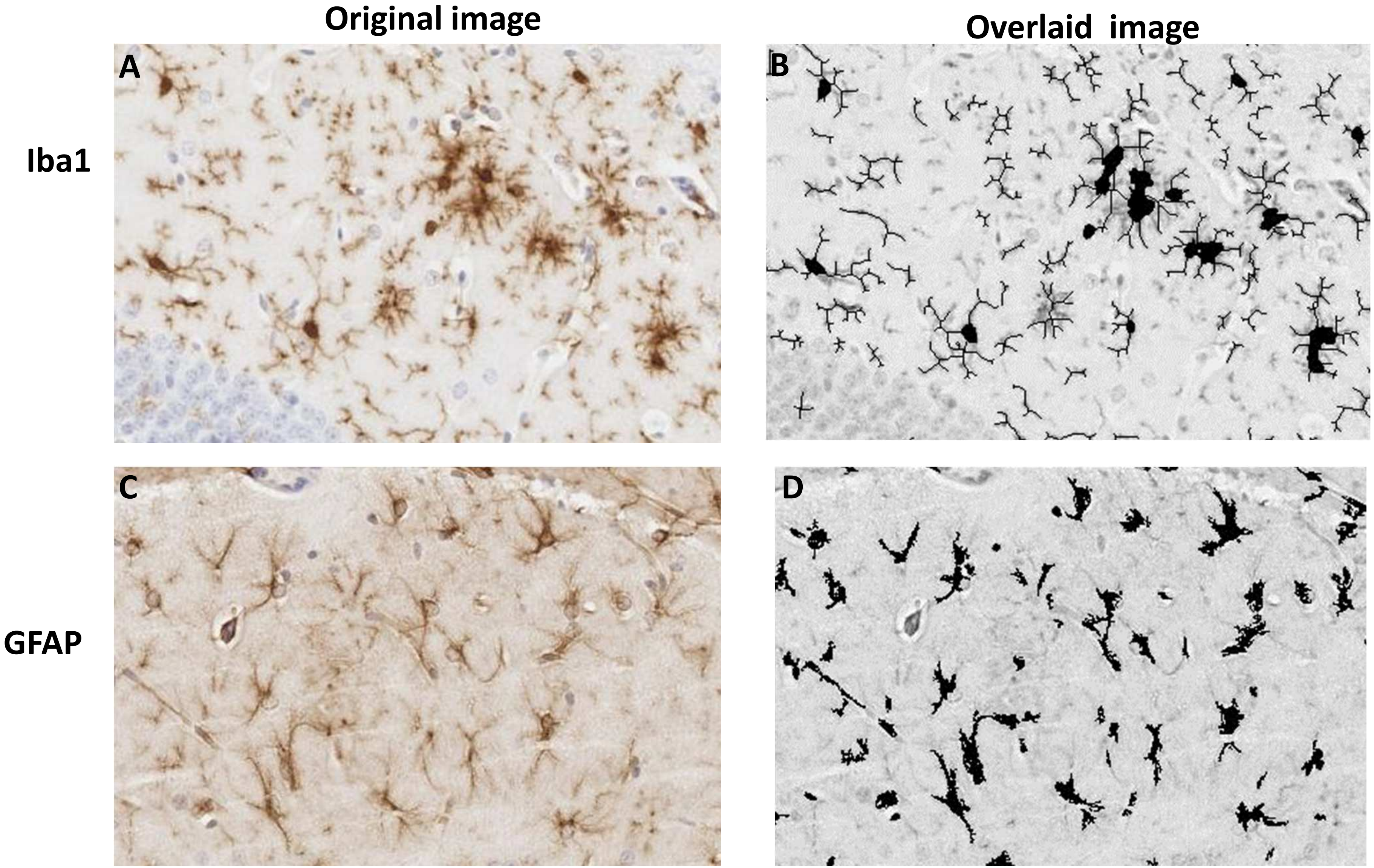

Suppl. Fig. 2: Histology based quantification of glial cells. Illustrative examples of extracted Iba1 positive microglia and microglial processes (A, B) and GFAP positive astrocytes (C,D), extracted using a custom made semi-automated segmentation tool. For extraction of morphometric features, a region of interest (ROI) was drawn on the digitized histology images at 20x magnification using custom made software (Matlab), outlining the region covered by the MRS spectroscopy voxel (2x2 mm2, extending over the hippocampus and thalamus). For feature recognition (using custom made software programmed in Matlab), soma were first identified in the ROIs on histological images by blurring with an average filter of adjacent pixels and thresholding adapted for uneven background staining. All images were inspected and corrected manually to avoid artifacts.

	Covariate	Genotype	Treatment	Time	Genotype X Treatment	Genotype X Time	Treatment X Time	Genotype X Treatment X Time
**MRS**	**Baseline levels**							
**GABA**	F(1,29)=3.76<br>p=0.06	F(1,29)=1.25<br>p=0.27	F(1,29)=0.05<br>p=0.81	F(4,120)=2.79<br>**p=0.029**	F(1,29)=1.44<br>p=0.24	F(4,120)=0.7<br>p=0.59	F(4,120)=0.47<br>p=0.76	F(4,120)=0.48<br>p=0.75
**Glu**	F(1,29)=18.46<br>**p=0.0002**	F(1,29)=1.98<br>p=0.17	F(1,29)=2.13<br>p=0.15	F(4,120)=1.45<br>p=0.22	F(1,29)=7.77<br>**p=0.009**	F(4,120)=1.67<br>p=0.16	F(4,120)=0.88<br>p=0.48	F(4,120)=1.05<br>p=0.38
**Glx**	F(1,29)=9.2<br>**p=0.005**	F(1,29)=0.16<br>p=0.69	F(1,29)=0.78<br>p=0.38	F(4,120)=6.66<br>**p<0.001**	F(1,29)=2.3<br>p=0.14	F(4,120)=3.27<br>**p=0.01**	F(4,120)=1.17<br>p=0.33	F(4,120)=0.47<br>p=0.76
**mI**	F(1,29)=1.19<br>p=0.28	F(1,29)=0.58<br>p=0.45	F(1,29)=0.18<br>p=0.68	F(4,120)=1.4<br>p=0.24	F(1,29)=1.47<br>p=0.23	F(4,120)=0.97<br>p=0.42	F(4,120)=0.33<br>p=0.86	F(4,120)=2.87<br>**p=0.026**
**ML9**	F(1,29)=40.61<br>**p<0.001**	F(1,29)=2.00<br>p=0.17	F(1,29)=5.65<br>**p=0.024**	F(4,120)=2.78<br>**p=0.03**	F(1,29)=5.39<br>**p=0.027**	F(4,120)=2.84<br>**p=0.027**	F(4,120)=3.37<br>**p=0.012**	F(4,120)=1.82<br>p=0.13
**Taurine**	F(1,29)=2.51<br>p=0.12	F(1,29)=0.76<br>p=0.39	F(1,29)=0.08<br>p=0.78	F(4,120)=18.24<br>**p<0.001**	F(1,29)=2.95<br>p=0.10	F(4,120)=1.31<br>p=0.27	F(4,120)=1.65<br>p=0.16	F(4,120)=1.42<br>p=0.23
**tCho**	F(1,29)=0.74<br>p=0.39	F(1,29)=3.67<br>p=0.065	F(1,29)=1.55<br>p=0.22	F(4,120)=15.19<br>**p<0.001**	F(1,29)=0.01<br>p=0.91	F(4,120)=2.37<br>p=0.06	F(4,120)=0.50<br>p=0.74	F(4,120)=0.31<br>p=0.87
**tCr**	F(1,29)=9.2<br>**p=0.005**	F(1,29)=0.16<br>p=0.69	F(1,29)=0.78<br>p=0.38	F(4,120)=6.66<br>**p<0.001**	F(1,29)=2.3<br>p=0.14	F(4,120)=3.27<br>**p=0.01**	F(4,120)=1.17<br>p=0.33	F(4,120)=0.47<br>p=0.76
**tNAA**	F(1,29)=7.56<br>**p=0.01**	F(1,29)=1.61<br>p=0.21	F(1,29)=0.26<br>p=0.61	F(4,120)=0.29<br>p=0.88	F(1,29)=4.17<br>**p=0.05**	F(4,120)=3.56<br>**p=0.009**	F(4,120)=0.28<br>p=0.89	F(4,120)=1.79<br>p=0.13
**Histology**<br>***Iba1***	**Number of microglial clusters**							
**% area stained**	F(1,29)=29.3<br>**p<0.0001**	F(1,29)=8.06<br>**p=0.008**	F(1,29)=6.01<br>**p=0.02**	N/A	F(1,29)=0.53<br>p=0.47	N/A	N/A	N/A
**Cells/mm^2**	F(1,29)=27.06<br>**p<0.0001**	F(1,29)=2.87<br>p=0.10	F(1,29)=0.11<br>p=0.74	N/A	F(1,29)=0.04<br>p=0.85	N/A	N/A	N/A
**Soma size**	F(1,29)=27.2<br>**p<0.0001**	F(1,29)=9.82<br>**p=0.004**	F(1,29)=14.18<br>**p=0.0008**	N/A	F(1,29)=2.6<br>p=0.12	N/A	N/A	N/A
GFAP								
**% area stained**	F(1,29)=0.08<br>p=0.77	F(1,29)=0.06<br>p=0.80	F(1,29)=0.01<br>p=0.93	N/A	F(1,29)=1.46<br>p=0.24	N/A	N/A	N/A

Suppl. Table 1: Statistical analysis of metabolite concentration and histological changes

	Baseline	1 hour post injection	2 hours post injection	3 hours post injection	4 hours post injection
**Glu**					
WT-PBS	0.221 ± 0.002	0.224 ± 0.002	0.229 ± 0.002	0.223 ± 0.003	0.229 ± 0.004
WT-LPS	0.217 ± 0.003	0.218 ± 0.003	0.218 ± 0.003	0.215 ± 0.002	0.217 ± 0.002
APP/PS1-PBS	0.221 ± 0.003	0.218 ± 0.002	0.218 ± 0.003	0.218 ± 0.002	0.222 ± 0.003
APP/PS1-LPS	0.222 ± 0.003	0.219 ± 0.003	0.221 ± 0.004	0.224 ± 0.004	0.222 ± 0.003
**Glx**					
WT-PBS	0.312 ± 0.002	0.328 ± 0.002	0.331 ± 0.003	0.321 ± 0.003	0.335 ± 0.004
WT-LPS	0.320 ± 0.003	0.320 ± 0.003	0.323 ± 0.002	0.321 ± 0.003	0.327 ± 0.005
APP/PS1-PBS	0.319 ± 0.004	0.324 ± 0.004	0.324 ± 0.003	0.326 ± 0.003	0.326 ± 0.004
APP/PS1-LPS	0.324 ± 0.004	0.327 ± 0.004	0.329 ± 0.005	0.333 ± 0.005	0.330 ± 0.005
**Ins**					
WT-PBS	0.116 ± 0.009	0.119 ± 0.003	0.114 ± 0.004	0.115 ± 0.004	0.117 ± 0.005
WT-LPS	0.116 ± 0.005	0.115 ± 0.005	0.116 ± 0.006	0.115 ± 0.006	0.115 ± 0.005
APP/PS1-PBS	0.112 ± 0.003	0.110 ± 0.009	0.113 ± 0.004	0.112 ± 0.003	0.113 ± 0.003
APP/PS1-LPS	0.110 ± 0.002	0.115 ± 0.003	0.109 ± 0.004	0.113 ± 0.002	0.117 ± 0.004
**ML9**					
WT-PBS	0.161 ± 0.004	0.158 ± 0.004	0.161 ± 0.005	0.167 ± 0.003	0.155 ± 0.006
WT-LPS	0.161 ± 0.011	0.173 ± 0.005	0.175 ± 0.007	0.183 ± 0.005	0.181 ± 0.003
APP/PS1-PBS	0.169 ± 0.018	0.189 ± 0.020	0.157 ± 0.006	0.158 ± 0.008	0.151 ± 0.010
APP/PS1-LPS	0.165 ± 0.003	0.167 ± 0.008	0.162 ± 0.005	0.173 ± 0.008	0.164 ± 0.007
**Tau**					
WT-PBS	0.194 ± 0.004	0.190 ± 0.004	0.188 ± 0.004	0.188 ± 0.005	0.183 ± 0.004
WT-LPS	0.191 ± 0.009	0.193 ± 0.008	0.187 ± 0.009	0.187 ± 0.009	0.181 ± 0.009
APP/PS1-PBS	0.204 ± 0.005	0.199 ± 0.005	0.199 ± 0.007	0.200 ± 0.005	0.194 ± 0.005
APP/PS1-LPS	0.201 ± 0.004	0.194 ± 0.003	0.197 ± 0.004	0.190 ± 0.002	0.180 ± 0.005
**tCho**					
WT-PBS	0.041 ± 0.001	0.038 ± 0.001	0.037 ± 0.001	0.038 ± 0.001	0.037 ± 0.001
WT-LPS	0.043 ± 0.001	0.041 ± 0.002	0.041 ± 0.002	0.040 ± 0.002	0.040 ± 0.002
APP/PS1-PBS	0.043 ± 0.001	0.042 ± 0.002	0.041 ± 0.002	0.040 ± 0.002	0.041 ± 0.001
APP/PS1-LPS	0.043 ± 0.001	0.042 ± 0.001	0.041 ± 0.001	0.040 ± 0.001	0.042 ± 0.001
**tCr**					
WT-PBS	0.194 ± 0.002	0.193 ± 0.001	0.197 ± 0.002	0.198 ± 0.001	0.196 ± 0.002
WT-LPS	0.197 ± 0.001	0.197 ± 0.002	0.199 ± 0.003	0.201 ± 0.002	0.201 ± 0.002
APP/PS1-PBS	0.191 ± 0.003	0.191 ± 0.002	0.192 ± 0.003	0.192 ± 0.001	0.194 ± 0.002
APP/PS1-LPS	0.189 ± 0.001	0.191 ± 0.002	0.194 ± 0.002	0.193 ± 0.002	0.198 ± 0.002
**tNAA**					
WT-PBS	0.163 ± 0.003	0.158 ± 0.003	0.158 ± 0.002	0.165 ± 0.002	0.157 ± 0.002
WT-LPS	0.159 ± 0.006	0.160 ± 0.005	0.161 ± 0.005	0.165 ± 0.006	0.161 ± 0.006
APP/PS1-PBS	0.155 ± 0.003	0.160 ± 0.005	0.159 ± 0.002	0.153 ± 0.007	0.159 ± 0.004
APP/PS1-LPS	0.155 ± 0.003	0.152 ± 0.002	0.151 ± 0.004	0.151 ± 0.003	0.154 ± 0.003

Suppl. Table 2. Mean ± SEM metabolite concentrations, expressed as ratios to the sum of selected metabolites, of WT and APP/PS1 mice treated with LPS or its vehicle PBS at baseline and after injection.
